# Clonal Variation in the Bark Chemical Properties of Hybrid Aspen: Potential for Added Value Chemicals

**DOI:** 10.3390/molecules25194403

**Published:** 2020-09-25

**Authors:** Pasi Korkalo, Risto Korpinen, Egbert Beuker, Tytti Sarjala, Jarkko Hellström, Janne Kaseva, Ulla Lassi, Tuula Jyske

**Affiliations:** 1 Production Systems, Natural Resources Institute Finland, Ounasjoentie 6, 96200 Rovaniemi, Finland; 2 Production Systems, Natural Resources Institute Finland, Tietotie 2, 02150 Espoo, Finland; risto.korpinen@luke.fi; 3 Production Systems, Natural Resources Institute Finland, Vipusenkuja 5, 57200 Savonlinna, Finland; egbert.beuker@luke.fi; 4 Production Systems, Natural Resources Institute Finland, Kaironiementie 15, 39700 Parkano, Finland; tytti.sarjala@luke.fi; 5 Production Systems, Natural Resources Institute Finland, Myllytie 1, 31600 Jokioinen, Finland; jarkko.hellstrom@luke.fi; 6 Natural Resources, Natural Resources Institute Finland, Tietotie 4, 31600 Jokioinen, Finland; janne.kaseva@luke.fi; 7 Research Unit of Sustainable Chemistry, University of Oulu, FI-90570 Oulu, Finland; ulla.lassi@oulu.fi

**Keywords:** hybrid aspen, clonal variation, bark, cascade, extractive, lignocellulosic biomass, suberic acid, condensed tannin, antioxidative capacity

## Abstract

This study aims to promote comprehensive utilization of woody biomass by providing a knowledgebase on the utility of aspen bark as a new alternative source for fossil-based chemicals. The research focused on the analysis of clonal variation in: (1) major chemical components, i.e., hemicelluloses, cellulose, and lignin; (2) extraneous materials, i.e., bark extractives, and suberic acid; (3) condensed tannins content and composition; and (4) screening differences in antioxidative properties and total phenolic content of hot water extracts and ethanol-water extracts of hybrid aspen bark. Results of this study, the discovery of clonal variation in utilizable chemicals, pave the way for further research on added-value potential of under-utilized hybrid aspen and its bark. Clonal variation was found in notable part of chemicals with potential for utilization. Based on the results, an appropriate bark raw material can be selected for tailored processing, thus improving the resource efficiency. The results also indicate that by applying cascade processing concepts, bark chemical substances could be more efficiently utilized with more environmentally friendly methods.

## 1. Introduction

The chemical industry is still heavily reliant on the use of fossil raw materials and a great need for alternative, sustainable ones exists. Lignocellulosic biomasses have received much scientific interest as renewable substitutes for different chemical industry applications, and thus the need for faster production of woody biomass is growing globally. This topic is timely also in Finland, where the rotation periods of commercially important tree species exceed over 70–80 years. One possible way to boost the production and availability of wood-based raw materials is to breed fast growing tree species designed for the chemical industry’s application purposes.

Hybrid aspen (*Populus tremula* L. × *tremuloides* Michx.) is one of the fastest growing tree species in Finland. On fertile sites in southern Finland, the yields may reach up to 20 m^3^ ha^−1^ per year in about 25-year rotations [1]. The species show excellent coppice vigor that enables even higher second rotation growth [2]. High-yield capacity and chemical properties of hybrid aspen indicate its promising potential as raw material for biochemicals and green energy [3,4]. Hybrid aspen could be an alternative for Norway spruce, which is presently predominantly used in reforestation of vigorous sites in Finland, but which faces severe problems due to *Heterobasidion* root rot disease [5] as well as with the spruce bark beetle (*Ips acuminatus* L.) [6], the risks of which are expected to increase with climate change.

Currently, bark of coniferous species is a major industrial by-product in the Nordic countries, being one of the most promising resource for added-value biochemical production. In Finland alone, the processes of forest industries use ca. 71 million m^3^ of round wood annually [7]. The amount of bark is approximately 10% of the round wood volume; thus, the ca. 7 million m^3^ of bark is produced as a by-product every year. This residue is still mainly used for energy production.

Like wood, aspen bark mainly consists of lignin, hemicellulose and cellulose, and earlier studies have shown that the bark of *Populus* species (*Salicaceae* family) are especially rich in extractable biochemical components. However, these phytochemicals have been poorly characterized [8]. Only a few have been extensively explored as pharmaceuticals (e.g., salicylic and benzoate-based drugs) and reached commercialization. Aspen bark extractives have potential to be utilized as natural source of antioxidants. *Populus tremuloides* Michx. bark hot water extract has reported to have greater antioxidative capacity than synthetic antioxidant butylated hydroxytoluene (BHT). With fractionating the bark crude water extracts with organic solvents, products antioxidative nature can be enhanced [9]. The lignin content has been reported to reach ca. 15% and the rest is composed of carbohydrate-derived compounds [10]. Additionally, the lipid content of aspen bark is 10% [11], but the lipid composition is still undetermined. The suberin (i.e., suberic acids) content of extractive free *Populus tremula* bark has been shown to be 37.9% [12]. Based on the existing literature, aspen bark is thus a very promising, still heavily under-utilized raw material.

Material science has great interest to develop environmentally friendly composite materials to substitute petroleum-based polymers, in which lignocellulosic biomasses have characteristics to be utilized in such applications. Aspen tree fiber material has been studied as filler in biocomposite and plastic-wood composite matrices. With designed compositions of polymeric ingredients, aspen tree materials showed potential to be used as part of composite materials [13,14,15].

For finding new value-added uses, more detailed clonal screening of the chemical diversity of bark is needed for aspen and poplar species and/or clones well adapted in the Finnish climate. This study aims to promote comprehensive utilization of woody biomass by providing a knowledgebase on aspen bark as a new alternative source for fossil-based chemicals. The research topics included analysis of clonal variation in: (1) major chemical components, i.e., hemicelluloses, cellulose, and lignin; (2) extraneous materials, i.e., bark extractives, and suberic acid; (3) condensed tannins content and composition; and (4) screening differences in antioxidative properties and total phenolic content of hot water extracts and ethanol-water extracts of hybrid aspen bark. For testing the hypothesis that no marked clonal differences in bark chemical properties exist, hybrid aspen trees representing three different clones with good growth and quality characteristics (based on earlier studies) were harvested, and bark samples at heights of 1.3 m and 5 m on the stem were analyzed.

## 2. Results

### 2.1. Clonal Variation in Major Chemical Components

**Figure 1:**
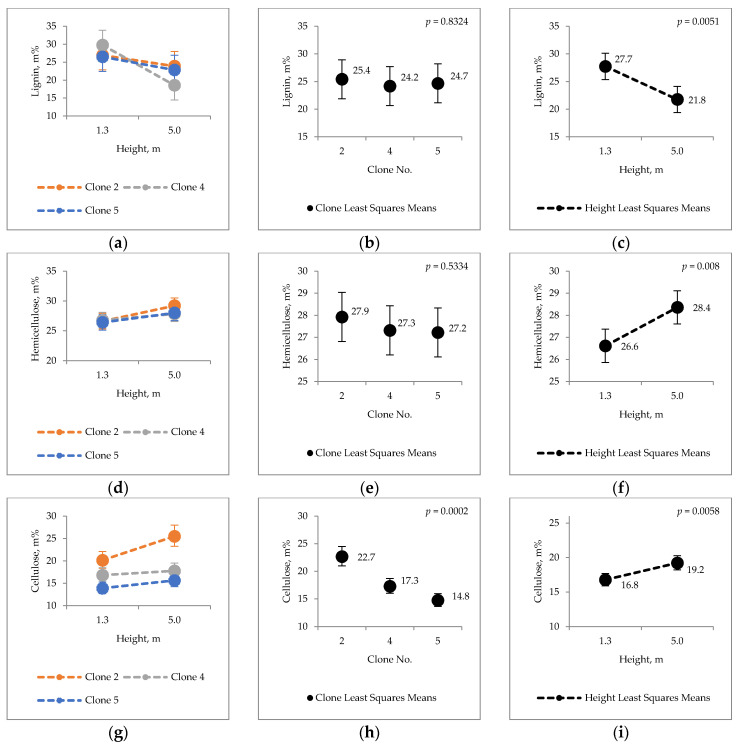
Content of lignin (**a**–**c**); hemicellulose (**d**–**f**); and cellulose (**g**–**i**) in the bark of hybrid aspen.

Analyte variations in the content and composition of major structural constituents of hybrid aspen bark are illustrated in Figure 1. The results show that the clonal averages for these chemical constituents of bark varied as follows: cellulose (14.8–22.7%), hemicellulose (26.6–28.4%) and lignin (21.8–27.7%). Hybrid aspen trunk height (i.e., sampling at 1.3 m and 5 m on the stem from ground) had a statistically significant effect on lignin, hemicellulose, and cellulose contents of bark material. Lignin content decreased 5.9 percentage points (pp) from the stem base (1.3 m) to the height of 5 m, but at same time, a 1.8 pp increase in hemicellulose content and 2.4 pp increase in cellulose content was found. Between the clones, a statistically significant difference was detected only in cellulose content, with the most pronounced difference of 53.4% being found between the clone 5 (14.8%) and clone 2 (22.7%) (clone 2 vs. 5 least squares means; *p* = 0.0002). Least squares means for height variable in the statistical analysis (Figure 1c,f,i) show that the total sums of major chemical components at the height of 1.3 m (71.1%) to 5 m (69.4%) change only 2.4%, while the proportions of lignin, cellulose and hemicellulose vary between the heights.

### 2.2. Extraneous Material

**Figure 2:**
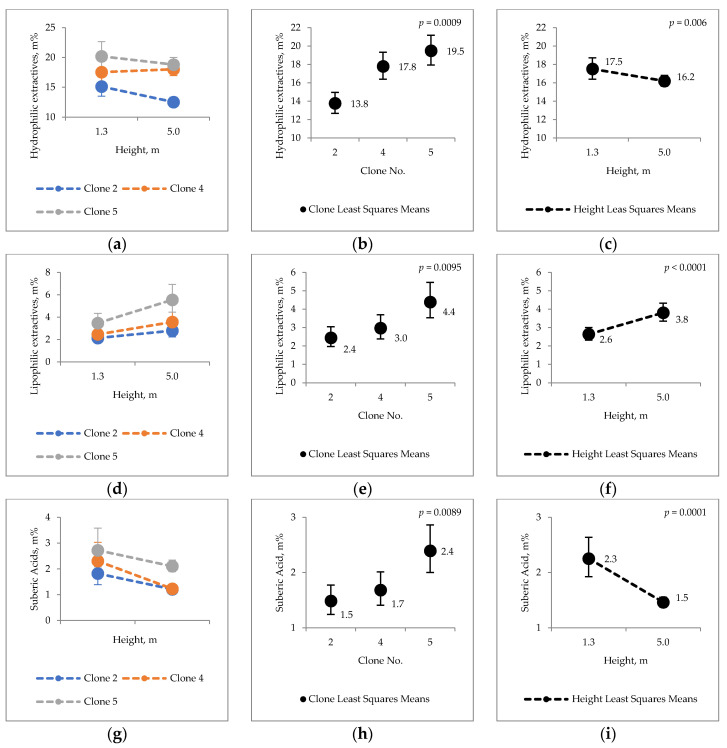

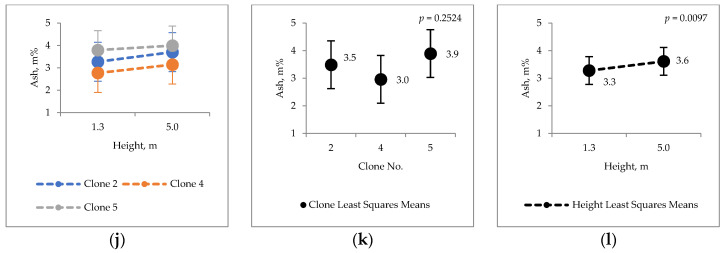
Content of (**a**–**c**) hydrophilic extractives; (**d**–**f**) lipophilic extractives; (**g**–**i**) suberic acid and (**j**–**l**) ash.

Analyzed extraneous materials included bark extractives and insoluble substances of suberic acid and ash. According to the results, the total amount of extractives in the bark of hybrid aspen varied between 16.2–23.9% dry weight (d.w.) (Figure 2). Statistically significant differences in hydrophilic extractives, lipophilic extractives, and suberic acid content were observed between the clones and between trunk heights (Figure 2). Both lipophilic and hydrophilic extractives, as well as suberin acid content, showed the highest levels in clone 5 and the lowest ones in clone 2. The content of hydrophilic extractives and lipophilic extractives in the bark of different clones varied between 13.8–19.5% d.w. (hydrophilic) and 2.4–4.4% d.w. (lipophilic), respectively. As calculated from the total extractive content, the proportion of lipophilic substances was prominently lower than that of hydrophilic ones. Yield of lipophilic extractives was notably higher (83.3%) in the clone 2 than that in clone 5 (clone least squares means; 2 vs. 5, *p* = 0.0086) (Figure 2e). Suberic acid content was found 60% greater in clone 5 than in clone 2 (clone least squares means 2 vs. 5; *p* = 0.0087) (Figure 2h).

Statistically evaluating, trunk height also had a significant effect on the amount of bark extractives and suberic acid. Following analyte content percent changes were found when comparing trunk height of 1.3 m to that of 5 m: a 7.4% decrease in hydrophilic extractives (Figure 2c), 46.2% increase in lipophilic extractives (Figure 2f) and 34.8% decrease in suberic acids (Figure 2i).

Ash content of hybrid aspen bark materials reached ca. 3–4% d.w. and showed statistical variations between heights, but no differences were found between the clones (Figure 2j–l).

### 2.3. Condensed Tannins

**Figure 3:**
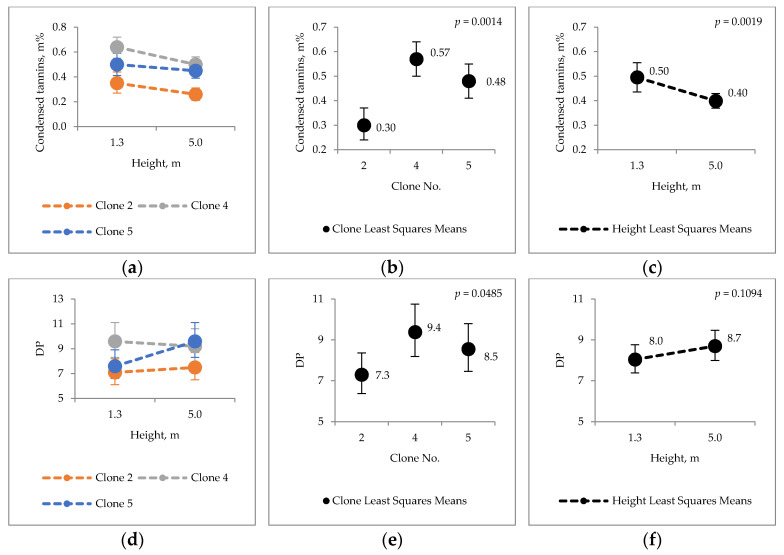

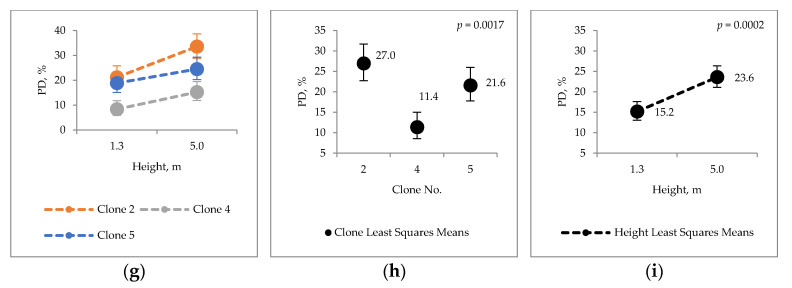
Hybrid aspen clone bark; (**a**–**c**) condensed tannins (CT) contents (**d**–**f**) average degree of polymerization (DP) for total flavan-3-ols and percentage value of prodelphinidins (PD, %), i.e., (epi)gallocatechin subunits in condensed tannins (**g**–**i**).

Total flavan-3-ols (proanthocyanidins, i.e., condensed tannins + monomeric flavan-3-ols) in aspen samples were measured by thioacidolysis. (Epi)catechin and (epi)gallocatechin structural units were detected, i.e., condensed tannins in clones were a mixture of procyanidins (PC) and prodelphinidins (PD). In results (Figure 3), only percentage share of PD is illustrated from the sum of PD and PC units. Degree of polymerization (DP) is the number of monomer units in the polymeric chain structure. The content of condensed tannins in the bark of hybrid aspen clones was ca. 0.30–0.57% d.w., variating between the studied clones and heights of 1.3 m and 5 m.

The degree of polymerization of tannins had statistically significant variations between the clones, and the proportions of PD and PC varied between heights. No clone × height interaction was found for CT, DP of PD/PC unit ratios.

### 2.4. Bark Hot Water and Ethanol Extracts Antioxidative Capacity and Total Phenol Contents

Bark materials antioxidative properties were characterized from hot water extracts (HWE) and 70% ethanol aq. (EtOH) extracts by using of ORAC, FRAP and H_2_O_2_ SCAV (scavenging) assays. The content of total phenolics was also analyzed from both extracts.

**Figure 4:**
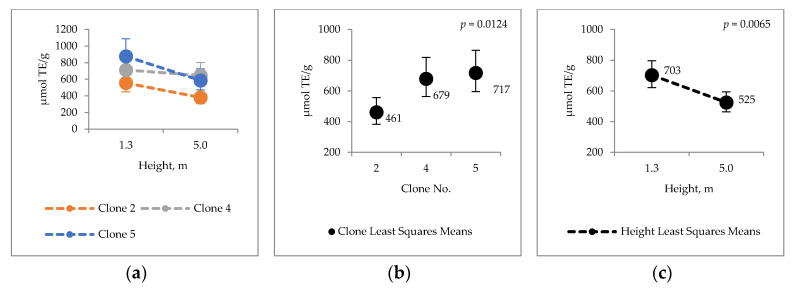

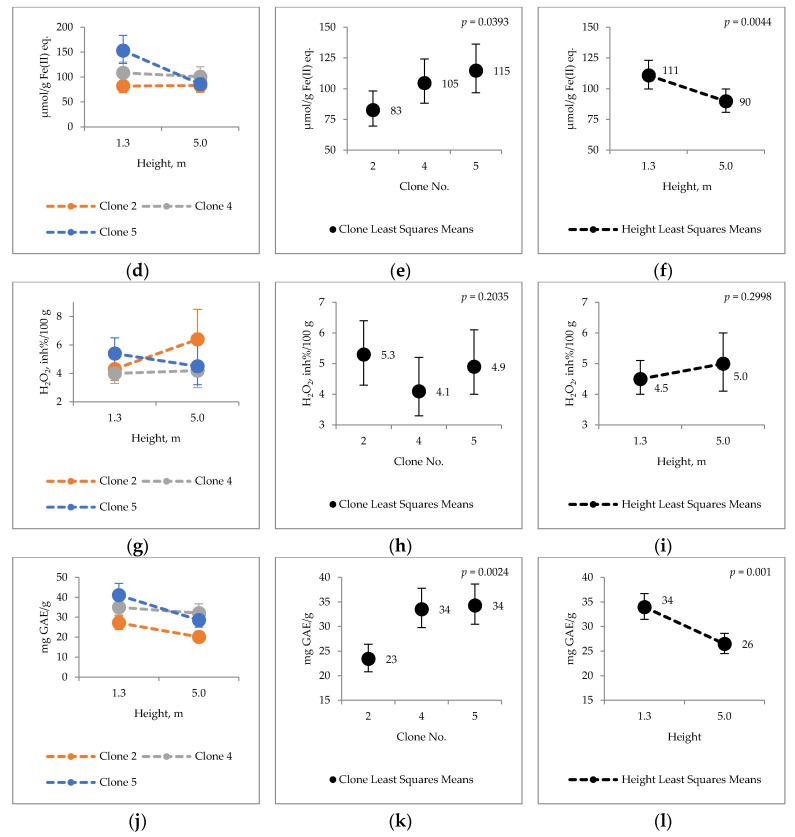
Hot water extract antioxidative capacity (**a**–**c**) ORAC µmol TE/g; (**d**–**f**) FRAP µmol/g Fe(II) eq.; (**g**–**i**) H_2_O_2_ scavenging H_2_O_2_ inh % per 100 g; (**j**–**l**) Total phenolic content mg GAE/g.

The antioxidative capacity of water extracts of hybrid aspen bark (Figure 4) was found variating as follows: ORAC 461–717 µmol TE/g; FRAP 83–115 µmol/g FE(II) eq. and H_2_O_2_ SCAV 4.1–5.3 inh%/100 g. The total phenol content varied between 23–34 mg GAE (gallic acid equivalence)/g.

**Figure 5:**
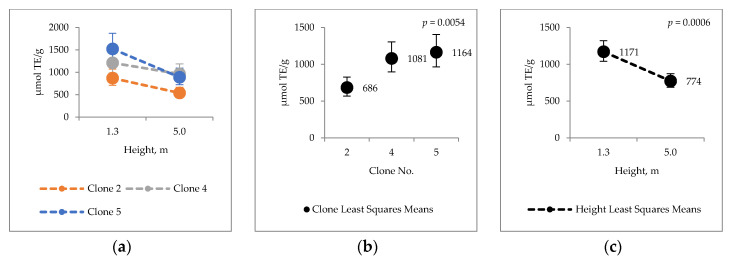

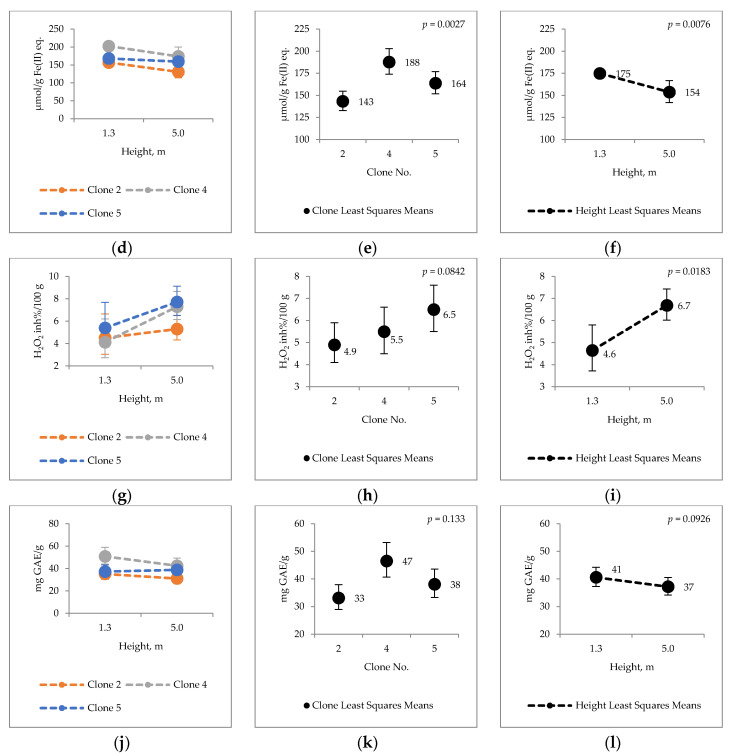
Ethanol extract antioxidative capacity (**a**–**c**) ORAC µmol TE/g; (**d**–**f**) FRAP µmol/g Fe(II) eq.; (**g**–**i**) H_2_O_2_ scavenging H_2_O_2_ inh % per 100 g; (**j**–**l**) Total phenolic content mg GAE/g.

The corresponding values antioxidative capacity for ethanol extracts 686–1171 µmol TE/g; 143–188 µmol/g FE(II) eq. and 4.9–6.7 inh%/100 g. The total phenolic content in ethanol extracts was 33–47 mg/g GAE (Figure 5).

Total phenol (HWE) content and antioxidative activity of water extracts measured by ORAC and FRAP tests varied statistically significantly between the clones and heights. ORAC and FRAP values for ethanol extracts variated between the clones and heights, but H_2_O_2_ SCAV had variations only between the heights. Furthermore, significant clone × height interactions were found for antioxidative capacity of hot water extracts analyzed by FRAP (*p* = 0.0047) and H_2_O_2_ SCAV (*p* = 0.0639) (Figure 4d,g).

## 3. Discussion

### 3.1. Major Chemical Components of Hybrid Aspen Bark

In the view of raw material chemical utilization, the results showed that the major chemical components of hybrid aspen bark were notably lower in cellulose content, but were in line with lignin and hemicellulose typically reported for xylem (i.e., wood) in hardwood species (Figure 1; [16], pp. 51–72). In general, wood cellulose content is estimated to be 40–45%, hemicellulose 20–30%, and lignin 20–25% in hardwood species ([16], pp. 51–72).

From the viewpoint of current wood utilization, cellulose is mainly used in the paper industry but has also great characteristics for nanocomposite applications [17]. Lignin is a major by-product from pulp and papermaking, but only a small fraction is collected for further high-added value purposes, even though it possesses possibilities to be utilized as chemical constituent in e.g., epoxy resins, foams, dispersants, adhesives and various polymer chemistry applications [18,19]. Hemicellulose can be utilized for producing gels, films and adhesives, but also has many application possibilities in the food and pharmaceutical industries [20]. Also, structural polysaccharides of hemicelluloses can be hydrolyzed into monomers; e.g., the most abundant sugar, xylose, can be catalytically converted into valuable furfural [21].

Hemicellulose and cellulose structural polysaccharides can be hydrolyzed into valuable products of monomeric sugar mixture units and be further converted into platform chemicals [22,23]. Chemical process has impact on the end-product quality [22,23], but also raw material characteristics determine its usability in various chemical applications.

Besides polymer chemistry obtained from lignocellulosic biomasses, woody side-stream mass can be converted via torrefaction procedures into more energy dense states, in which carbon rich solid residue consists mainly of lignin and cellulose fractions [24]. In mild torrefaction process, mainly hemicellulose content degrades into gas and distillates [24]. Torrefied willow distillate has found to have characteristics to be potentially utilized as pesticide [25]. Lower contents of hemicellulose, cellulose and lignin in hybrid aspen bark may have effects on solid products of torrefaction, especially if torrefication is carried out without any pre-extraction methods to remove extractive contents.

In the case of raw material upgrade via clonal breeding design, no practically important clonal differences were found in this study; however, dividing and sorting the logs into lower and upper parts, the chemical quality of obtained bark may be improved, thus also increasing the value of the material, e.g., bark with higher levels of valuable chemical components. Regarding the potential interests on cellulose structural sugar glucose, the most valuable raw material is obtained from the clone 2, stem height of 5 m. Therefore, selected clonal breeds or separated lower/upper log fractions may offer raw materials with tailored/optimized properties for improved value acquisition for different applications. The differences in the studied analytes between the heights are mainly due to differences in bark age (i.e., younger bark on the upper and older bark at the lower stem section). Thus, for optimizing the chemical quality of hybrid aspen bark, stand/tree age in harvesting operations should be considered. In this study, the growth rate of the studied hybrid aspen trees of the three different clones did not differ (*p* > 0.05 for D1.3 m, D5 m, height and base of the living crown). Thus, detected differences in chemical characteristics of bark may be of genetic origin. However, more research is needed to generalize these findings, as only three trees per clone were analyzed, with no replicates from different growing sites or geographical regions.

### 3.2. Extraneous Material of Hybrid Aspen Bark

Hybrid aspen bark extractive amounts of this study yielded 16.2–23.9% d.w. When compared to the previous research showing that the content bark extractives is ca. 20–40% d.w., the extractive content of hybrid aspen bark found in this study was on the lower end of the reported variation [16] (pp. 112, 113).

Hybrid aspen bark suberin content was found to vary between 1.5–2.4% d.w., where clone 5 had the highest amounts at the lower half of trunk. In this study, the suberin content of hybrid aspen bark was similar to that of birch bark (5.9% d.w.) [26], but only when compared with the bulk bark, including both the inner and outer layers of bark. The suberic acid content of cork cells in the outer layers of bark can reach up to 20–40% [16] (p. 113). Birch suberin rich outer bark layer can be more easily removed than that of hybrid aspen bark. In practice, by separating inner and outer bark layers of birch bark, suberin rich outer layer material is obtained and can yield up to 45% d.w. of suberin in bark [27]. Hybrid aspen bark would most likely be collected as whole bulk mass, where suberin yield would remain lower. In utilization purposes, suberin has been studied as an environmentally friendly substitutive for coating chemicals, where suberic acids from birch outer bark have been found to function well when applied as water repellent coatings [28].

Extractable tannins have a long history in leather production but could also serve as viable options in many biomaterial applications such as adhesives, resins, and foams [29,30]. Norwegian spruce bark has been studied in a carbon foams application; the condensed tannin content of spruce bark has been reported to be 3% and be mainly composed of (epi)catechins and substantially lower proportion of (epi)gallocatechins [30]. As compared to spruce bark, the content of condensed tannins in hybrid aspen bark was notable lower, namely 0.30–0.57% of d.w. The observed amount in this study was also prominently lower than the findings by Lindroth and Whang [31], who have found condensed tannin content of 18% for leaf materials of *Populus tremuloides* Michx. clonal trees [31].

### 3.3. Antioxidative Properties of Hybrid Aspen Bark

The antioxidative capacities of hybrid aspen bark found in this study are in line with the findings reported by Kähkönen, et al. [32], who found that the total phenol content of bark in *Populus tremula* was 32.1 mg GAE/g [32]. In contrast, higher bark phenolic contents have been reported for other fast-growing tree species, e.g., willow *Salix caprea* (75.5 mg GAE/g) and silver willow (*Salix alba*) (58.6 mg GAE/g) [32]. On other hand, bark phenol content of hybrid aspen appears to be higher than those observed in typical Nordic wild berries, namely bilberry (*Vaccinium myrtillus*, 29.7 mg GAE/g); cowberry (*Vaccinium vitis-idaea*, 24.9 mg GAE/g) and cloudberry (*Rubus chamaemorus*, 16.2 mg GAE/g) [32]. Moreover, the ORAC values obtained in this study for bark of hybrid aspen appeared to be high when compared to ORAC values reported for bilberry of 282.3 µmol TE/g of dry matter [33].

Berries are prominent non-timber forest products in Finland and generally considered as important source of health beneficial phenolic substances. As compared above, hybrid aspen bark chemical characteristics indicate that bark mass possesses great antioxidative capacity and could be viable, potential source of phenolic compounds as well. This study also highlights that high antioxidative capacity was obtained via hot water extraction only, and therefore valuable chemicals are possible to extract without the use of organic solvents. Botanical extracts with antioxidative activity and high phenol contents are of interest for nutraceutical industry, as well as for other health related applications such as cosmetics and dermatological products [34]. Plants antioxidative phytochemicals can offer a natural alternative for synthetic phenolic antioxidants [35]. In Fennoscandia, wild berries are known as valuable source of antioxidative biomasses and considered as one important non-timber forest product [36,37], but also from wood material, willow (same Salicaceae family as populous trees) has shown great potential to be utilized as an phenolic antioxidant source with great antioxidative properties [32].

## 4. Materials and Methods

### 4.1. Sample Trees

Three trees from each of three hybrid aspen (*Populus tremula* L. *× P. tremuloides* Michx.), clones (no. 2, 4 and 5) were randomly selected from a random-block designed field trial of the Natural Resource Institute Finland (Luke) in Punkaharju, in southeastern Finland. For each clone each tree was selected from a different block. The trial was established in May 2002 with 3 × 3 m plant spacing and the sample trees were felled in February 2018. For this study, from each tree two sample discs were collected from height 1.3 m and 5 m. Characteristics of the sample trees are presented in Table 1.

### 4.2. Statistical Analysis

All dependent variables were analyzed by generalized linear model (GLM) having clone (2, 3 and 4), height (1.3 m and 5 m), and their interaction as fixed effects. Three replicates were used leading to sample size of 18 per variable. Correlation between heights within same experimental unit was taken into account using a heterogeneous or a homogeneous compound symmetry structure. The first one allows unequal variances for both heights and the latter handles those as equal, respectively. The most suitable information criterion (AICc) for small sample size was used in comparison of the covariance structures, and a likelihood test was used as a side to help the decision making [39]. The normality of residuals was studied by graphically from multiple residual plots and founded adequate.

Only lignin, hemicellulose and ash were analyzed with the assumption of normal distribution. GLM with the assumption of lognormal (with an identity link) or Gamma distribution (with a log link) was used, when distributions of dependent variables were highly skewed. The assumption of beta distribution (with a logit link) was used for percentages. Restricted maximum likelihood estimation method (REML) was used for normal and lognormal distributions and residual pseudo-likelihood estimation method (REPL) for gamma and beta distributions, respectively [40].

Six cross-comparisons (e.g., clone 2 in height 1.3 m vs. clone 4 in height 5 m) of the interaction term were excluded to minimize the amount of pairwise comparisons. The stepdown method of Westfall [41] was used for pairwise comparisons of means (significance level of α = 0.05), which is known to be one of the most effective in cases when the design is balanced. Kenward–Roger method [42] was used for calculating the degrees of freedom.

The analyses were performed using the GLIMMIX procedure of the SAS Enterprise Guide 7.15 (SAS Institute Inc., Cary, NC, USA).

Differences in the chemical properties between stem heights and clones were calculated as percentage difference (Equation (1)). Compared values are expressed in Figure 1, Figure 2, Figure 3, Figure 4 and Figure 5.

(1) $$\frac{\left(A-B\right)}{B}\;\times \;100\%$$

### 4.3. Wood Analysis

#### 4.3.1. Sample Preparation

Bark samples for chemical analysis were manually separated from freshly cut aspen tree trunks. Bark mass contained both inner and outer bark layers. Bark materials were first freeze-dried and then grinded by a Pulverisette cutting mill (Type 15.903, Fritsch, Idar-Oberstein, Germany) with 2 mm sieve cassette. Powder with particle-size of <2 mm was used for *cellulose*, *hemicellulose*, *lignin* and *suberic acid* content analysis. First, samples were extracted with accelerated solvent extraction (ASE-350, Dionex, Sunnyvale, CA, USA) method in order to remove lipophilic and hydrophilic extractives. To increase the effectiveness of lignin and suberic acid extraction, extract free samples were further milled into fine powder with an IKA A 10 analytical mill (Kinematica, Littau/Luzern, Switzerland) equipped with a cooling unit (−20 °C).

Fine bark powder for the assays of *antioxidative properties* and analysis of *total phenol content* and *condensed tannin* were prepared by first grinding the samples by using a Fritsch Pulverisette mill and then by an A 10 universal batch mill (IKA, Staufen, Germany). Powdered samples’ particle-size was estimated with test sieve set of 1.25, 0.5, 0.2, and 0.0063 mm sieves from one bark sample after IKA A 10 grind. Particle-size distribution estimates are expressed in Table 2. Moisture content of the samples was checked with moisture analyzer (Moisture analyzer MLB 50-3N, KERN & Sohn GmbH, Balingen, Germany).

#### 4.3.2. Lipohilic and Hydrophilic Extractives

Lipophilic and hydrophilic extractives was gravimetrically analyzed with accelerated solvent extraction (Dionex ASE-350) method. For extractions, bark samples were loaded to stainless steel cylinders weighing approximately 7.5–8.5 g each. Bark samples lipophilic extractives was extracted with hexane 90 °C, 3 × 15 min cycles and hydrophilic extractives with 95% acetone (aq) 100 °C, 3 × 15 min cycles. Each lipophilic and hydrophilic extractives end volume was adjusted to 50 mL with extraction solvents. Extractive content was determinated gravimetrically from 6 mL of extract by drying it under nitrogen gas flow in 40 °C temperature-controlled bath and measuring the weight of dry solid residues.

#### 4.3.3. Suberic Acids

Suberic acids were determined from 2 g of extract-free samples. Samples were weighed out into 50 mL seal tight test tubes and 25 mL of 3% potassium hydroxide ethanol solution (KOH *w*/*v* EtOH) was added. Sample tubes was kept for 2 h in a 70 °C temperature-controlled bath. Suberic acid extract was separated from bark residues by vacuum filtration. Extract-free bark residue was washed with water and oven dried at 105 °C for 24 h for later lignin analysis. For suberic acid GC-MS analysis, samples were prepared by pipetting 0.3 mL of extract into a 15 mL glass test tube and diluting the extract with 3 mL of water. Two drops of bromocresol green was added as a pH indicator and extracts acidified (pH < 3.8) with 0.25 M sulphuric acid (H_2_SO_4_). Two mL of internal standard C21:0/betunilol 0.2 mg/mL in methyl *tert*-butyl ether (MTBE) was added and thoroughly shaken. Suberic acids were separated from extracts with liquid-liquid separation by adding again 2 mL of MTBE, vortexing thoroughly and letting phases to settle, collecting MTBE phase into a new 15 mL glass test tube and repeating the liquid-liquid extraction two more times with 3 mL of MTBE. Finally, the collected suberic acid MTBE extract was washed with 2 mL of water. Extracts were dried under a nitrogen flow in 40 °C temperature-controlled bath. Next 150 µL of silylating agent (1:4:1 mix of N,O-bis(trimethylsilyl)trifluoroacetamide (BSTFA):chlorotrimethylsilane (TMCS, Merck KGaA, Darmstadt, Germany):Pyridine) was added into test tubes and put into a 70 °C temperature-controlled bath for 45 min. Ready samples was transferred to GC-MS vials and analyzed. Suberic acid compounds were recognized by comparison with MS library values and the sum of total suberic acid content was determined by means of an internal C21:0 standard [43].

#### 4.3.4. Lignin

Lignin content was determined from previously collected suberic acid and extract free bark samples as a sum of an acid soluble and insoluble (Klason) lignin. Duplicate oven dried samples (200 mg) were prepared by weighing them out into closable 50 mL glass test tubes and adding 2 mL of 72% sulphuric acid (H_2_SO_4_). After mixing thoroughly, samples were incubated in 30 °C for 1 h. After incubation, solid residues and extracts was transferred into 100 mL closable pressure and heat resistant glassware by washing samples with 56 mL of water having solid residue in 4% sulphuric acid (aq) in the end. Samples were placed in an autoclave for 1 h in 120–125 °C. After the autoclaving process, samples were allowed to cool down to ambient temperature and acid soluble and insoluble samples were separated from each other by vacuum filtration. Filtered and washed solids were dried in 105 °C for 24 h and determined gravimetrically as an acid insoluble Klason lignin. Filter collected solution containing acid soluble lignin was measured by UV-Vis spectroscopy at 240 nm wavelength and lignin content was calculated by reference to the corresponding Laboratory Analytical Procedure [44].

#### 4.3.5. Cellulose

Cellulose content was determined by acid hydrolysis from extract free bark samples. Ten mg samples were weighed out into closable 10 mL glass test tubes as duplicates. Two cellulose standards were made in the same way. A small glass ball was inserted to every test tube to help subsequent mixing. Next 200 µL of 72% sulphuric acid (H_2_SO_4_) was added and allowed to react for 2 h. Then 0.5 mL of water was mixed into the samples and left to react for 4 h. Finally, 6 mL of water was mixed into the samples that were left to stand at room temperature overnight. The next day, test tubes were put into an autoclave for 1 h at 120–125 °C. After the sample test tubes had cooled down to ambient temperature, 2 drops of bromocresol green pH indicator was added. Small amounts of barium carbonate were added carefully, and the mixture was vortexed thoroughly after every addition until a blue color appeared. One mL of D-sorbitol internal standard (250 mg/50 mL H_2_O) was added and after mixing the samples were centrifuged. Supernatants were collected and dried under a nitrogen flow in 40 °C. After the samples were dried, the residual solvent was removed with the help of a 40 °C vacuum oven. Samples were silylated by adding in order 150 µL pyridine, 150 µL of 1,1,1,3,3,3-hexamethyldisilazane (HMDS, Sigma-Aldrich Chemie GmbH, Steinheim, Germany) and finally 70 µL of trimethylsilyl chloride (TMCS, Merck KGaA, Darmstadt, Germany). After careful mixing, samples were left to react overnight at room temperature. The next day, the clear liquid phase was collected and analyzed by GC-FID [43].

#### 4.3.6. Hemicellulose

Hemicellulose content and structural sugars were determined with acid methanolysis. Analyses were done as duplicate by weighing out 8–12 mg of extract free bark samples into pear shaped flasks. Standard samples were made from 1.0 mL of monosaccharide solvent mix containing arabinose, glucose, glucuronic acid, galactose, galacturonic acid, mannose, rhamnose and xylose (1.0 mg/mL each). Two mL of methanolysis reagent 2M HCl (anhydrous MeOH) was added and vortexed thoroughly before placed into oven for 5 h under 100 °C. After vials cooled down, samples were neutralized by adding 80 µL of pyridine. One mL of inner standard sorbitol 0.1 mg/mL + resorcinol 0.1 mg/mL (MeOH) was added in each sample and vortexed. After solutions settled down, 1 mL of clear phase was collected and dried out under nitrogen flow in 40 °C temperature-controlled bath. Dry sample residues were silylated by adding 150 µL pyridine, 150 µL HMDS disilazane and 70 µL TMCS in order. Samples were left overnight in room temperature and settled sample solutions clear phase was carefully collected and analyzed by GC-FID [45].

### 4.4. Condensed Tannins

Condensed tannins (CT, proanthocyanidins) were determined by HPLC after thiolytic degradation. Samples were weighed (20–30 mg) into 1.5 mL Eppendorf vials and 1 mL of depolymerization reagent (3 g cysteamine dissolved in 56 mL of methanol acidified with 4 mL of 13 M HCl) was added. The vials were sealed, vigorously mixed (vortexer) and incubated for 60 min at 65 °C. During the incubation the samples were vortexed few seconds every 15 min. After 60 min the samples were transferred into an ice bath to stop the reaction. Samples were filtrated into HPLC vials and analyzed on an Agilent 1290 Infinity UHPLC device equipped with a Zorbax Eclipse Plus C18 column (Agilent Technologies, Santa Clara, CA, USA, 50 × 2.1 mm i.d., 1.8 μm). The binary mobile phase consisted of 0.5% formic acid (aq) and acetonitrile. Elution was started with 2% acetonitrile, isocratically for 2 min, followed by a linear gradient to 5% in 3 min, to 15% in 7 min, to 20% in 3 min, to 35% in 5 min, to 90% in 1 min, and back to the starting point in 2 min. The post-time was 2 min before the next injection. The flow rate was 0.5 mL/min and injection volume 2 μL. Elution was monitored by diode array detection (DAD; λ1 = 270 nm, λ2 = 280 nm) and fluorescence detection (FLD; λ_ex_ = 275 nm, λ_em_ = 324 nm). CT degradation products, i.e., free flavan-3-ols (terminal units) and their cysteaminyl derivatives (extension units), were quantified using external standards of catechin, epicatechin, gallocatechin, epigallocatechin, and thiolyzed procyanidin B2. Cysteamine, catechin, epicatechin, gallocatechin, and epigallocatechin were purchased from Sigma-Aldrich Finland Oy (Espoo, Finland). Procyanidin B2 was obtained from Extrasynthese (Lyon, France).

### 4.5. Antioxidative Properties and Total Phenol Content

Samples of dried aspen bark were extracted (1:10) for the bioactivity testing with hot distilled water or 70% ethanol. Bark powder was weighed in 15 mL test tubes and boiling H_2_O (or 70% ethanol (aq)) was added to the tube, then vortexed for 2 min, incubated for 15 min at RT after which vortexed for 2 min, and centrifuged for 10 min at 8400 rpm (Sigma 2-16KL). After centrifugation the supernatant was removed to a clean tube and kept at +4 °C until tested.

To screen the antioxidative properties of the aspen bark fractions, four assays to cover different antioxidant mechanisms were used. The measurements were performed on 96-microplate format with a multimode microplate reader (Varioskan Flash, Thermo Scientific, Waltham, MA, USA). Detailed descriptions of the testing protocols of ORAC, FRAP and SCAV are described in the paper of Vaario et al. [46].

#### 4.5.1. Ferric Reducing Antioxidant Power (FRAP)

Single electron transfer (SET)-based FRAP (ferric reducing antioxidant power) assay measures the ability of an antioxidant to reduce ferric (FeIII) to ferrous (FeII) ion FeSO_4_·7H_2_O was used as a standard and four different dilutions of the samples were used to fit them to the standard curve.

The samples were mixed with 20 mM FeCl_3_·6H_2_O and 10 mM 2,4,6-tris(2-pyridyl)-s-triazine (TPTZ) in 300 mM acetate buffer pH 3.6. The assay was run with three technical replicates and the absorbance was measured at 594 nm after the formation of ferrous-tripyridyltriazine complex in the reaction mixture. Reagents: FeSO_4_·7H_2_O (Sigma-Aldrich Chemie GmbH), l(+)-ascorbic acid (VWR Chemicals, Radnor, PA, USA), 2,4,6-tris(2-pyridyl)-s-triazine (TPTZ, Sigma-Aldrich Chemie GmbH, St. Louis, MO, USA) [47].

#### 4.5.2. Oxygen Radical Absorbance Capacity

Hydrogen atom transfer-based (HAT) the Oxygen Radical Absorbance Capacity (ORAC) assay measures the oxidative dissociation of fluorescein at the presence of peroxyl radicals, which causes reduction in the fluorescence signal. The antioxidative ability of an extract or a compound is measured as the inhibition of the fluorescein breakdown. The method used here was modified to 96-well format from those described by Huang et al. [48] and Prior et al. [49] with two technical replicates of each sample on the plate.

The sample in 0.075 M phosphate buffer pH 7.5 was mixed with 8.16 × 10^−5^ mM fluorescein and 2,2′-azobis(2-methylpropionamidine) dihydrochloride). A series of twenty dilutions was used for each sample to adjust the sample concentration to the standard curve. Trolox was used as a standard and the results are expressed as Trolox ((±)-6-Hydroxy-2,5,7,8,-tetramethylchromane-2-carboxylic acid, a vitamin E analog) equivalents (µmol/L TE). Reagents: Phosphate buffer pH 7.5 (Merck), fluorescein (Sigma-Aldrich Chemie GmbH, St. Louis, MO, USA), 2,2′-azobis(2-methylpropionamidine) dihydrochloride (Sigma-Aldrich Chemie GmbH, St. Louis, MO, USA). Trolox ((±)-6-hydroxy-2,5,7,8,-tetramethylchromane-2-carboxylic acid, vitamin E analog) (Sigma-Aldrich Chemie GmbH, St. Louis, MO, USA).

#### 4.5.3. H_2_O_2_ Scavenging Assay

H_2_O_2_ scavenging assay, based on transition metal chelation, was modified from Hazra et al. [50] and Jiang et al. [51] with microplate reader in 96-well format with four technical replicates on each plate. In the assay the formation of ferric-xylenol orange complex indicates the ability of the sample to scavenge H_2_O_2_ and prevent the oxidation of Fe(II) to Fe(III). Each sample was measured using the extract (1:10) without dilutions and the result is expressed as inhibition percentage (%) of Fe(II) oxidation to Fe(III).

In the assay the sample is mixed with an aliquot of 2 mM H_2_O_2_, 2.56 mM ammonium iron (II) sulphate·6H_2_O and 111 µM xylenol orange disodium salt and after 30 min incubation, the absorbance of ferric-xylenol orange complex at 560 nm was measured.) Sodium pyruvate (Sigma-Aldrich Chemie GmbH, St. Louis, MO, USA) was used as a reference compound. Reagents: H_2_O_2_ (Merck KGaA, Darmstadt, Germany), ammonium iron (II) sulphate·6H_2_O (BDH Prolabo, Dubai, UAE), xylenol orange disodium salt (Sigma-Aldrich Chemie GmbH, St. Louis, MO, USA), sodium pyruvate (Sigma-Aldrich Chemie GmbH, St. Louis, MO, USA).

#### 4.5.4. Folin-Ciocalteu Assay

Folin-Ciocalteu assay [52,53,54] was used to analyze the total phenolic content in the extracts. The samples were mixed with Folin-Ciocalteu reagent and Na_2_CO_3_ and absorbance measured at 750 nm with gallic acid as a reference compound. The results are expressed as gallic acid equivalents per 1 g (mg GAE/g). Reagents: Na_2_CO_3_ (Merck KGaA), gallic acid (Sigma-Aldrich Chemie GmbH, St. Louis, MO, USA), ethanol (Altia Industrial, Helsinki, Finland), Folin-Ciocalteu reagents (Merck KGaA, Darmstadt, Germany).

### 4.6. Ash Content

Ash content was determined with a modified TAPPI protocol [55]. One g of fine-grained samples was weight out and dried in 105 °C for 24 h. Bone dry powder was weighed and placed into the furnace. The ashing furnace temperature program was set to rise 80 °C per hour from ambient to 270 °C, then 220 °C per hour to 470 °C and finally 100 °C per hour to the target temperature of 540 °C and kept for 180 min. After the furnace had cooled down, the remaining incombustibles were determined gravimetrically and calculated as ash content.

## 5. Conclusions

The results of this study pave the way for further research on added-value potential of under-utilized hybrid aspen and its bark. Clonal variation was found in notable part of studied utilizable chemicals. Based on the results, an appropriate bark raw material can be selected for tailored processing, thus improving the resource efficiency. Promising results for antioxidative properties of hot water extractives of bark also indicate that by applying cascade processing concepts, bark chemical substances could be fully utilized without producing new waste-streams. For example, as bark material is directed into torrefaction processes for solid material upgrade, an environmentally friendly water extraction method can be viable option to collect valuable extracts before the extractive free bark material ends into thermochemical treatment for lignocellulosic biomass utilization. Hybrid aspen bark solid material upgrade and cascade processing to collect extractable valuables remains as a topic for further studies.

## Figures and Tables

**Table 1:** Characteristics of the sample trees

Clone No.	National Register Id ^1^	H (dm) ^2^	LTL (dm) ^3^	D 1.3 (mm) ^4^	D 5.0 (mm) ^5^
2	C05-99-24	188 ± 18	57 ± 5	153 ± 12	129 ± 12
4	C05-99-34	189 ± 21	52 ± 19	141 ± 12	118 ± 13
5	C05-99-14	180 ± 38	52 ± 18	124 ± 16	104 ± 28

^1^ The national list of approved basic forest reproductive material, kept by the Finnish Food Authority [38], ^2^ H dm = tree height, ^3^ LTL = Height of Living Treetop Line measured from ground level, ^4,5^ 1.3 m and 5.0 m sample discs cross length measured in north-south orientation.

**Table 2:** Pulverized bark sample particle-size distribution.

Particle-Size Distribution	
<0.063 mm	11.7%
0.063–0.2 mm	20.1%
0.2–0.5 mm	42.3%
0.5–1.25 mm	25.2%
1.25–2.0 mm	0.6%
